# Whole Genome Sequence Analysis of Multidrug-Resistant *Staphylococcus aureus* and *Staphylococcus pseudintermedius* Isolated from Superficial Pyoderma in Dogs and Cats

**DOI:** 10.3390/antibiotics14070643

**Published:** 2025-06-25

**Authors:** Phirabhat Saengsawang, Ruedeechanok Tanonkaew, Rungruedee Kimseng, Veeranoot Nissapatorn, Phitchayapak Wintachai, Manuel J. Rodríguez-Ortega, Watcharapong Mitsuwan

**Affiliations:** 1 Akkhraratchakumari Veterinary College, Walailak University, Nakhon Si Thammarat 80160, Thailand; phirabhat.sa@wu.ac.th (P.S.); ruedeechanok.ta@mail.wu.ac.th (R.T.); 2 One Health Research Center, Walailak University, Nakhon Si Thammarat 80160, Thailand; 3 Sanders-Brown Center on Aging, College of Medicine, University of Kentucky, Lexington, KY 40506, USA; kim.rrd@uky.edu; 4 Futuristic Science Research Center, School of Science, Walailak University, Nakhon Si Thammarat 80160, Thailand; veeranoot.ni@wu.ac.th; 5 School of Science, Walailak University, Thasala, Nakhon Si Thammarat 80161, Thailand; phitchayapak.wi@wu.ac.th; 6 Departamento de Bioquímica y Biología Molecular, Universidad de Córdoba, Campus de Excelencia Internacional CeiA3, 14071 Córdoba, Spain; mjrodriguez@uco.es; 7 Center of Excellence in Innovation of Essential Oil and Bioactive Compounds, Walailak University, Nakhon Si Thammarat 80160, Thailand

**Keywords:** antibiotic resistance, multidrug resistance, pyoderma, *Staphylococcus aureus*, *Staphylococcus pseudintermedius*, whole genome sequence

## Abstract

**Background**: Pyoderma is a superficial bacterial infection that is considered the formation of pus-containing lesions on the skin occurring in animals. Staphylococci, including *Staphylococcus aureus* and *Staphylococcus pseudintermedius*, that cause pyoderma in pet animals is a global health concern. The objectives of this study were to investigate antibiotic-resistant staphylococci isolated from pyoderma in dogs and cats and to analyse whole genome sequences of multidrug-resistant (MDR) staphylococci. **Methods**: A total of 56 pyoderma swabbing samples from 42 dogs and 14 cats located in Southern Thailand was collected to isolate staphylococci. Antibiotic susceptibility and antibiotic-resistant genes of staphylococcal isolates were investigated. Furthermore, the representative MDR isolates were investigated using whole genome sequence analysis. **Results**: 61 isolates were identified as staphylococci, which can be classified into 12 different species, mostly including 13 *S. intermedius* (13.26%), 13 *S. saprophyticus* (13.26%), 8 *S. sciuri* (8.16%), and *Staphylococcus cohnii* (8.16%). Remarkably, the main pyoderma-causing species that were isolated in this study were *S. aureus* (5.10%) and *S. pseudintermedius* (3.06%). Most staphylococci were resistant to penicillin G (30%), and the *blaZ* gene was found to be the highest prevalence of the resistance genes. Both MDR-*S. aureus* WU1-1 and MDR-*S. pseudintermedius* WU48-1 carried capsule-related genes as main virulence factor genes. Interestingly, MDR-*S. pseudintermedius* WU48-1 was resistant to seven antibiotic classes, which simultaneously carried *blaZ*, *mecA*, *aac*, *dfrK*, *aph3*, and *tetM*. Genes related to antibiotic efflux were the highest proportion of the mechanism found in both representatives. Remarkably, SCCmec cassette genes were found in both isolates; however, the *mecA* gene was found only in MDR-*S. pseudintermedius* WU48-1. In addition, these were mostly carried by macrolide- and tetracycline-resistance genes. Mobile gene transfer and horizontal gene transfer events frequently contain genes involved in the antibiotic target alteration mechanism. **Conclusions**: This study found that MDR staphylococci, especially *S. aureus* and *S. pseudintermedius*, are important in animals and owners in terms of One Health concern. The information on whole genome sequences of these MDR staphylococci, particularly antimicrobial resistance genes, mobile genetic elements, and horizontal gene transfer events, can help to understand gene transmission and be applied for antibiotic resistance surveillance in veterinary medicine.

## 1. Introduction

Pyoderma is a superficial bacterial infection that involves the formation of pus-containing lesions on the skin occurring in animals [1]. In dogs and cats, pyoderma causes crusting, pustules, irritation, and even hair loss [2], and contributes to the significant clinical abnormalities, including pruritus, pain, and inflammation [3]. The main cause of the inflammatory response in pyoderma is frequently related to bacterial infection, particularly pyogenic cocci [1,3]. Staphylococci are mostly grouped as a normal flora on animal skin, for instance, dogs [4], and play important roles as opportunistic pathogens such as *Staphylococcus aureus* [5], *Staphylococcus pseudintermedius* [6], and *Staphylococcus sciuri* [7]. *Staphylococcus aureus* is a well-known main cause of a variety of infectious diseases, mainly in humans and sometimes in animals, ranging from skin infections to life-threatening disorders [2]. In addition, *S. pseudintermedius* is a normal flora mostly on the skin of dogs and cats, which can cause pyoderma infection [8], particularly in unhealthy skin conditions such as dermatitis, allergy, or a damaged skin barrier. *Mammaliicoccus sciuri* (formerly *S. sciuri*), a coagulase-negative staphylococci, is also defined as an opportunistic pathogen that causes serious subcutaneous abscesses in animals and humans [7,9]. Interestingly, *S. aureus*, *S. pseudintermedius*, and *S. sciuri* have been reported as emerging zoonotic pathogens to humans by the interaction between owners and pets, especially infected animal wound contact [10]. Moreover, *S. aureus*, *S. pseudintermedius*, and *S. sciuri* are known to be common biofilm producers, which mostly induce antibiotic resistance [11,12]. Staphylococcal cells are protected in an exopolysaccharide or biofilm, which initially enhances pathogenicity by promoting bacterial adhesion to tissues and surfaces [13]. Biofilm protects the bacterial cells from host immune defences, as well as resistance to antibiotics [14]. Pyoderma caused by staphylococci infection in dogs and cats mostly occurs from an opportunistic infection [15,16].

Antimicrobial resistance (AMR) is regarded as a global public health issue [17]. Particularly in hospitals and communities, antibiotic resistance is widely spread to humans [18], and close contact with animals is one of the transmission routes for antibiotic-resistant bacteria [19]. Currently, One Health in general links AMR, specifically antibiotic resistance, to interactions between humans, animals, and the environment. The excessive use of antibiotics in these sectors, responsible for agriculture (environmental sector), veterinary medicine (animal sector), and human medicine (human sector), induces AMR as well as the spreading of antibiotic-resistant bacteria [20,21]. Methicillin-resistant *S. aureus* (MRSA) in animals and humans revealed a close genetic relationship [22], and the transmission of MRSA in humans might be from animals such as dogs and cats [23]. In addition, methicillin-resistant *S. pseudintermedius* (MRSP) represents a significant resistance species in both dogs and cats. Importantly, the transmission of the pathogens from superficial staphylococci-infected animals to the pet owner via direct contact is a public health issue, and this plays an important role in the mode of methicillin-resistant strain transmission from pets to humans [24]. Of this, the understanding of staphylococci, particularly *S. aureus* and *S. pseudintermedius*, can help complete the antibiotic resistance transmission chain between humans and animals.

Whole genome sequencing is an advanced, powerful technique that is used to explore microbial genomes. In the aspects of antibiotic resistance and virulence, most antibiotic resistance genes and genes related to virulent factors can be analysed from whole genome data. In addition, the information from whole-genome analysis would contribute to understanding antibiotic resistance mechanisms, gene transfer, and virulence of microorganisms. Furthermore, the information about the antibiotic resistance genes in genomes can be used for horizontal gene transfer events and mobile genetic element prediction, which helps to explain antibiotic resistance in staphylococci. Considering the advantages of whole genome sequencing (WGS) compared to PCR or phenotypic-related methods can explain antimicrobial resistance genes and virulence genes better than traditional techniques. Although PCR and phenotypic assays remain valuable tools for detecting established resistance and virulence markers, their scope is restricted to predefined targets, thereby limiting their capacity to identify novel or rare genetic elements. Conversely, WGS provides an unbiased, genome-wide perspective, enabling the comprehensive detection of both known and previously unrecognised antimicrobial resistance and virulence genes. Recently, the whole genome sequence technique was used to explore the pathogenesis of *S. pseudintermedius* isolated from canine pyoderma [25] and canine and human bacteraemia infections [26]. Several species of staphylococci have been completely sequenced; however, the information on antimicrobial genes and mechanisms is lacking and needs further investigation, especially in multidrug-resistant and methicillin-resistant strains. The sequencing of bacterial genomes reveals deep information on genes such as bacterial virulence factor-related genes and antimicrobial-related genes [25]. Therefore, the objective of this study was (1) to determine the antibiotic resistance of staphylococci isolated from superficial pyoderma and (2) to analyse whole genome sequences of MDR-staphylococci isolated from dogs and cats in Nakhon Si Thammarat, Southern Thailand.

## 2. Results

### 2.1. Isolation and Identification of Staphylococci

A total of 98 staphylococcal-like strains were isolated from pyoderma lesions in 42 dogs and 14 cats, according to the growth characteristics of mannitol salt agar (MSA) and the presence of Gram-positive staphylococci under the microscope (Table 1). However, non-staphylococci (37 isolates) grown in the tested medium were also identified. All isolates were further identified for their species using MALDI-TOF/MS analysis. Sixty-one isolates (62.24%) were identified as the genus *Staphylococcus* with 12 different species (Table 2). Of this, *S. intermedius* (13 isolates/13.26%) and *S. saprophyticus* (13 isolates/13.26%) were the main species detected in the wound, followed by *S. sciuri* (8 isolates/8.16%) and *S. cohnii* (8 isolates/8.16%), respectively. In this study, common pyoderma-causing agents, including *S. aureus* (4 isolates/4.08%) and *S. pseudintermedius* (3 isolates/3.06%), were detected in the wounds of the animals. Moreover, non-staphylococci microorganisms, including *Micrococcus luteus* (11 isolates/11.22%), *Brevibacterium casei* (7 isolates/7.14%), and *Candida tropicalis* (8 isolates/8.16%), were found from the collected samples.

### 2.2. Antibiotic Susceptibility and MDR Patterns

Antibiotic susceptibility of the isolated staphylococci was investigated on seven antibiotic classes with 10 antibiotics. The findings revealed that most staphylococci were resistant to penicillin G (30%), followed by ampicillin (23%), erythromycin (21%), and doxycycline (20%), respectively (Table 3). Most of the antibiotic-resistant strains were *S. aureus*, *S. pseudintermedius*, and *S. sciuri*. In this study, one isolate of methicillin-resistant *S. pseudintermedius* WU48-1 was identified by disc diffusion assay. Importantly, *S. pseudintermedius* WU48-1 was found to be resistant to nine tested antibiotics classified into seven antibiotic classes, including methicillin. The results revealed that 11 isolated bacteria showed multi-drug resistance with five different antibiotic resistance patterns (Table 4). The most prevalent MDR pattern was PEN-AMP-KAN-DOX, with a prevalence of 54.55%, followed by PEN-AMP-DOX-TRI (18.18%). Interestingly, the MDR pattern of methicillin-resistant *S. pseudintermedius* WU48-1, which is resistant to seven antibiotic classes associated with nine tested antibiotics, was PEN-AMP-MET-ERY-CHL-CIP-KAN-DOX-TRI.

### 2.3. Detection of Antibiotic-Resistant Genes

A total of 24 resistance genes were identified from 16 chosen isolates. The detection of the antibiotic-resistant genes in isolated staphylococci is presented in Table 5. The *blaZ* gene was found to have the highest prevalence of the resistance genes detected. All *S. aureus* isolates carried *blaZ*, which was associated with beta-lactam antibiotics. In addition, *aph3*, a gene associated with aminoglycoside resistance, was the second antibiotic-resistant gene detected in the tested isolates. Interestingly, *S. pseudintermedius* WU48-1, which resisted seven antibiotic classes, carried *blaZ*, *mecA*, *aac*, *dfrK*, *aph3*, and *tetM*. Only one methicillin-resistant *S. pseudintermedius* isolate was found; however, there was no isolated *S. aureus* carrying the *mecA* gene. Furthermore, this isolate was defined as methicillin-resistant *S. pseudintermedius* due to the presence of both a methicillin-resistant phenotype and the *mecA* gene.

### 2.4. Whole Genome Characteristics of S. aureus WU1-1 and S. pseudintermedius WU48-1

In this study, MDR-*S. aureus* WU1-1 and MDR-*S. pseudintermedius* WU48-1 were selected for their whole genome sequencing. As shown in Table 6, genome information of *S. aureus* WU1-1 and *S. pseudintermedius* WU48-1 revealed a total of 2.8 Mbp (32.5% GC content) and 2.7 Mbp (37.5% GC content), respectively. The annotation results showed that *S. aureus* WU1-1 contained 2814 genes and *S. pseudintermedius* WU48-1 contained 2625 genes. Among those genes, there were 2668 and 2475 protein-coding genes detected in *S. aureus* WU1-1 and *S. pseudintermedius* WU48-1, respectively.

### 2.5. Multilocus Sequence Typing of S. aureus WU1-1 and S. pseudintermedius WU48-1

**Figure 1:**
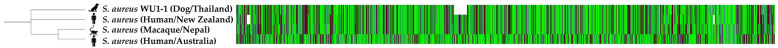
Core genome single-nucleotide polymorphism (cgSNP) analysis of whole-genome sequence relatedness of *S. aureus* strain WU1-1 with other strains of the same MLST types.

Only *S. aureus* WU1-1 revealed allele best matches for all loci, including allele 1 of the *arcC* locus, allele 1 of the *aroE* locus, allele 1 of the *glpF* locus, allele 1 of the *gmk* locus, allele 330 of the *pta* locus, allele 1 of the *tpi* locus, and allele 10 of the *yqiL* locus. Of this, the genome of isolated bacteria was ST2990, which was grouped in the clonal complex 1. In addition, the genome of *S. aureus* WU1-1 matched with the genome of other reported ST2990 from human wounds in Malaysia, human unspecified invasive disease in New Zealand, and another human unspecified invasive disease in Australia. The analysis of core genome single-nucleotide polymorphism (cgSNP) based on whole-genome sequencing data to compare the genomic relatedness of *S. aureus* strain WU1-1 with other strains of the same MLST type is presented in Figure 1. The cgSNP analysis of four ST2990 isolates was performed using 2,239,954 bp of the core genome. The mapping and alignment results indicated that our *S. aureus* WU1-1, isolated from a dog in Thailand, was highly similar to an isolate from a human in New Zealand. In contrast, a higher number of cgSNPs—approximately 1000—was observed between *S. aureus* ST2990 isolates from a macaque in Nepal and a human in Australia. The cgSNP variation of *S. aureus* WU1-1 was 0.045%, which was lower than that observed in the other ST2990 isolates. In addition, *S. pseudintermedius* WU48-1 was matched with a reported ST1386 in a dog from Sweden (allele 2 of *ack*, allele 9 of *cpn* 60, allele 3 of *fdh*, allele 1 of *pta*, allele 20 of *purA*, allele 2 of *sar*, and allele 2 of *tuf*). However, this strain was not matched to any clonal complex. 

### 2.6. Virulence Gene Determination in Whole Genome Sequences of S. aureus WU1-1 and S. pseudintermedius WU48-1

**Figure 2:**
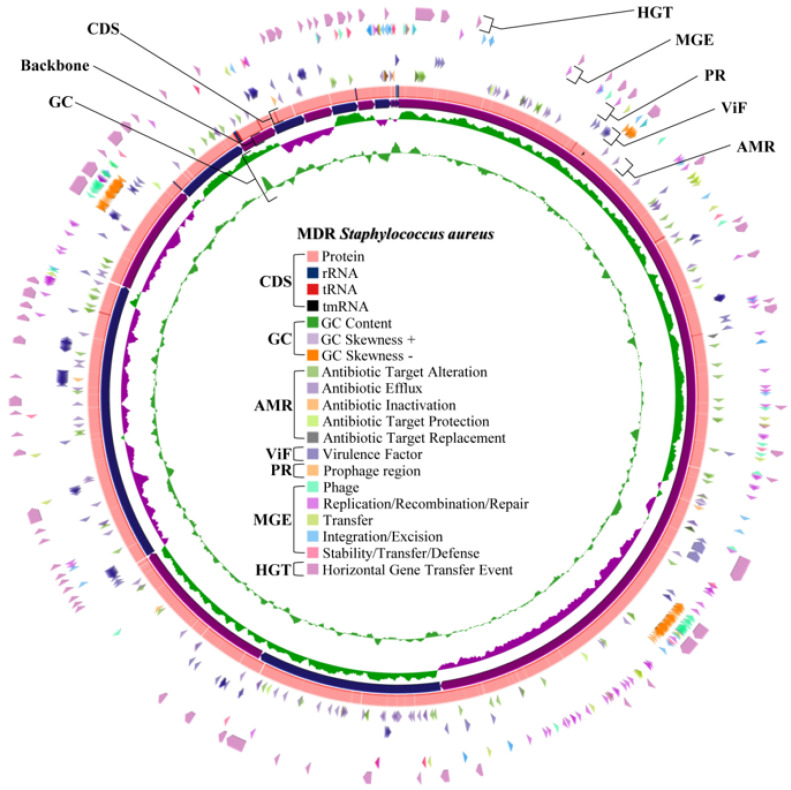
Whole genome mapping of multidrug resistance *S. aureus* WU1-1 (CDS; protein coding sequence, AMR; antimicrobial resistance gene, ViF; virulence factor related gene, PR; prophage region, MGE; mobile gene element, HGT; horizontal gene transfer).

**Figure 3:**
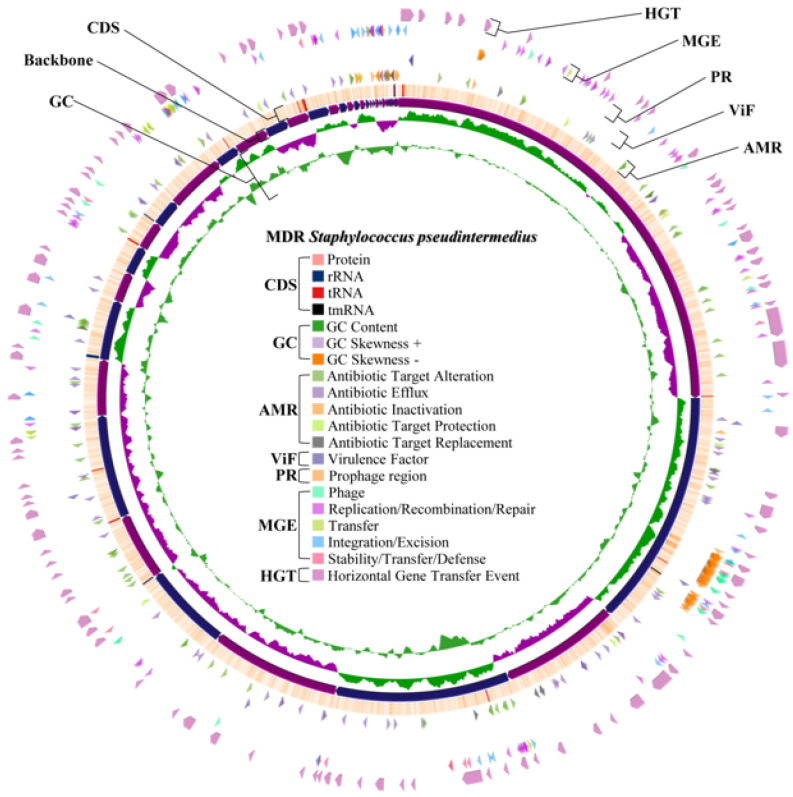
Whole genome mapping of multidrug resistance *S. pseudintermedius* WU48-1 (CDS, protein coding sequence; AMR, antimicrobial resistance gene; ViF, virulence factor related gene; PR, prophage region; MGE, mobile gene element; HGT, horizontal gene transfer).

Virulence gene prediction of both whole genomes was obtained from the VFDB, which revealed 128 virulence factor genes for *S. aureus* WU1-1 and 5 virulence factor genes for *S. pseudintermedius* WU48-1. The details of virulence-factor-related genes of both representative strains are presented in Table 6. All discovered virulence factors of each representative isolate were mapped in their draft whole genome (Figure 2 for *S. aureus* WU1-1 and Figure 3 for *S. pseudintermedius* WU48-1). The main virulent factors found in MDR *S. aureus* WU1-1 were capsule, staphyloferrin, fibronectin binding protein, exotoxin, haemolysin, and type VII secretion system. However, the number of virulent factors in *S. pseudintermedius* WU48-1 was relatively low. Interestingly, *S. aureus* WU1-1 was found to have nine exotoxins, which were related to the *set* gene family; nevertheless, there is no exotoxin reported in *S. pseudintermedius* WU48-1. A total of 8/9 *set* genes are located close together and near a gene transcribed as transposase, named the *tnp* gene. Interestingly, 6/9 *set* genes were found as horizontal gene transfer events. *S. aureus* produces several haemolysins that lyse erythrocytes. In addition, only *S. aureus* WU1-1 harboured seven haemolysin genes, including one alpha haemolysin gene (*hly*/*hla*), one beta haemolysin gene (*hlb*) at two different sites, three gamma haemolysin genes (*hlgA*, *hlgB*, and *hlgC*), and one delta haemolysin gene (*hld*).

### 2.7. Antimicrobial Resistance Gene in Whole Genome Sequence Determination of S. aureus WU1-1 and S. pseudintermedius WU48-1

In total, 173 genes and 170 genes were associated with antimicrobial resistance found in *S. aureus* WU1-1 and *S. pseudintermedius* WU48-1, respectively. Figure 2 and Figure 3 present the distribution of common antimicrobial resistance genes mapped in their draft whole genomes. Both representative isolates primarily contained genes related to the antibiotic efflux mechanism. Importantly, both isolates harboured genes in the SCCmec cassette (Table 6). The *mecA* gene and *mecR1* gene were found in methicillin-resistant *S. pseudintermedius* WU48-1, while the *mecC* gene, *mecI* gene, and *mecR1* gene were found in the genome of *S. aureus* WU1-1. All isolates contained the genes that were distributed for antibiotic efflux, antibiotic alteration, antibiotic target protection, antibiotic target replacement, and antibiotic inactivation. Mainly, antibiotic efflux and antibiotic target alteration were found as major resistance mechanisms of MDR staphylococci. Both *S. aureus* WU1-1 and *S. pseudintermedius* WU48-1 mostly carried antibiotic resistance genes related to macrolide and tetracycline classes.

### 2.8. Horizontal Gene Transfer and Mobile Genetic Element Prediction of Antimicrobial Resistance Genes in S. aureus WU1-1 and S. pseudintermedius WU48-1

**Figure 4:**
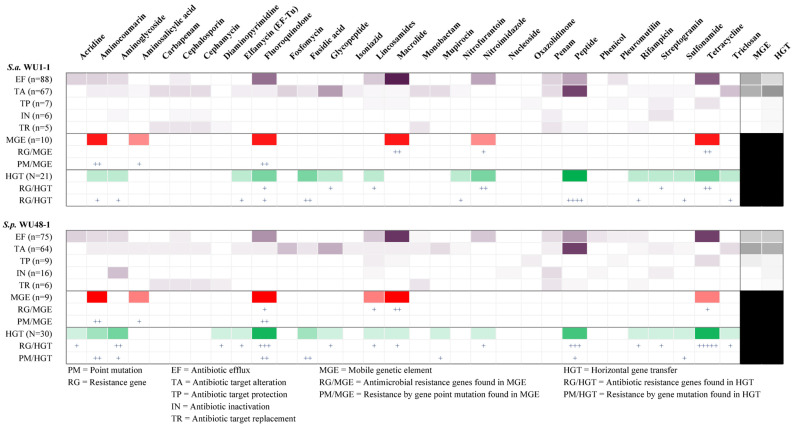
Heatmap of antimicrobial resistance genes classified by antimicrobial groups and resistance mechanisms of MDR-*S. aureus* WU1-1 and *S. pseudintermedius* WU48-1 (+ is represented for the number of genes in each category).

Antimicrobial resistance genes related to the efflux pump mechanism were found to be macrolide resistant in the highest proportion of genes in the whole genome of both *S. aureus* WU1-1 and *S. pseudintermedius* WU48-1. In addition, antimicrobial resistance genes related to tetracycline were found to be the second proportion of resistance gene in the efflux pump mechanism in both genomes. Mobile genetic elements (MGEs) and horizontal gene transfer events were the highest found in the antibiotic target alteration mechanism, next to the efflux pump mechanism. Moreover, genes related to aminocoumarin, fluoroquinolone, macrolide, and tetracycline resistances were mostly found as mobile gene elements in both *S. aureus* WU1-1 and *S. pseudintermedius* WU48-1. While genes related to the resistance of fluoroquinolone, peptides, and tetracycline were found to be horizontally transferred, other than in other groups. The heatmap of antimicrobial resistance genes classified by antimicrobial groups and resistance mechanisms is presented in Figure 4. However, the study of other resistance genes, such as aminocoumarin, fluoroquinolone, and tetracycline in the aspects of MGEs events, is less.

### 2.9. Prediction of the Prophage Insertion Region of S. aureus WU1-1 and S. pseudintermedius WU48-1

From the prediction, *S. aureus* WU1-1 was inserted by prophage DNA into three regions (Figure 2). Triavirus DNA was found as the insert at the first region of the *S. aureus* WU1-1 genome, while Dubowvirus inserted its DNA at the second and third regions of the genome. In addition, *S. pseudintermedius* WU48-1 revealed three prophage regions (Figure 3). DNA sequences of predicted prophage regions were additionally searched to confirm the type of bacteriophage in the NCBI virus database. Coventryvirus (*Staphylococcus* phage) DNA was found as inserted prophage DNA at the first region of the *S. pseudintermedius* WU48-1 genome. Moreover, Dubowvirus and Phietavirus (both are *Staphylococcus* phage) DNA were also found at the second and third regions of this bacterial genome, respectively. Even if each prophage region of both *S. aureus* WU1-1 and *S. pseudintermedius* WU48-1 contained different inserted DNA of the species of virus, the DNA of the virus of the predicted prophage was classified in the same class. Accordingly, all prophage regions were inserted with DNA of the bacteriophage classified in the Caudoviricetes class.

## 3. Discussion

Pyoderma caused by staphylococci is one of the severe morbidities in animals. The findings revealed that the most frequent bacteria found in the skin infections in both dogs and cats in Southern Thailand were coagulase-negative staphylococci. Nonetheless, coagulase-negative staphylococci were less pathogenic than coagulase-positive staphylococci like *S. aureus* and *S. pseudintermedius* [27]. Particularly, both *S. aureus* and *S. pseudintermedius* have been found as the main pathogens detected in Thailand [28,29,30], and it is widely recognised that the most causative agent that induces canine pyoderma is *S. pseudintermedius* [31]. *S. pseudintermedius*, previously classified in *S. intermedius* [32], is an opportunistic bacteria-inducing skin infection in both dogs and cats [33]. Interestingly, *S*. *pseudintermedius*, especially MDR strains, is a concern for humans due to its zoonotic potential through close contact between humans and pets [34]. Notably, the beta-lactam antibiotic group, including penicillin, has been reported as the most common occurrence in antibiotic resistance occurring in staphylococci isolated from dogs and cats [35,36]. In veterinary medicine, MDR *S. pseudintermedius,* as well as MRSP, have been problematic [37]. Besides, treatment of MRSP infections is becoming more difficult using the antimicrobials currently approved for veterinary use [38]. The transmission of antimicrobial-resistant bacteria has become a One Health issue since AMR bacteria can be transmitted between humans, animals, and the environment [10,14]. Due to the increasing popularity of household pets, the likelihood of close contact between animals and humans is rising [3], which may facilitate the cross-species transmission of AMR bacteria from close contact between pet owners and their pets [39,40,41]. According to previous reports, the prevalence of methicillin-resistant *S. pseudintermedius* in dogs in Southern Thailand was approximately 28.30% (15/53) [29], which is higher than our study. In addition, the occurrence of the *mecA* gene in staphylococci contrasted with the previous study in Thailand, which presented an extremely high rate of prevalence [29]. In the present, methicillin-resistant *S. pseudintermedius* is a common concern, especially in cases of skin infections in dogs, and this problem affects the selection of antibiotic treatment options in both human and veterinary medicine [38]. Moreover, this scenario has forced veterinarians to apply antimicrobials exclusively approved for human use, which the World Health Organization defines as critically important antimicrobials [42].

This study revealed whole genome sequencing analysis of MDR-*S. aureus* WU1-1 and MDR-*S. pseudintermedius* WU48-1. Several studies revealed the whole genome sequence of *S. aureus* and *S. pseudintermedius* isolated from canine pyoderma with insightful information on virulence factors and antibiotic resistance genes [25,26,43]. The finding genome size is similar to the previous report on whole-genome staphylococci isolated from animals [25]. In addition, the genomes of the *S. pseudintermedius* isolates from canine pyoderma were noticeably larger than those of isolates from healthy skin [25]. However, the samples were collected from dog pyoderma that were not compared to the samples from healthy dog skin in this area. Our results demonstrated that the main virulence factors found in MDR *S. aureus* WU1-1 were capsule, staphyloferrin, fibronectin-binding protein, exotoxin, haemolysin, and type VII secretion system. However, the number of virulence factors in *S. pseudintermedius* WU48-1 was relatively low, compared with *S. aureus* WU1-1. Genes related to the capsule are the most proportionate in the genomes. In general, the capsule was found to be the main virulence factor in both staphylococcus isolates. *S. aureus* synthesises a capsule that protects from attacking aspects of the immune system, with menadione probably involved in the synthesis [44]. The primary immunological evasion mechanism of the capsule is recognised as the ability to enhance the resistance to phagocytosis. In addition, capsules play an important role in the interference of antibody recognition against bacterial cell walls and complement activation blockage [45]. Of this, capsules of staphylococci mostly play an important role in immune attack interference, and the role of staphylococcal capsules in skin infections is not reported elsewhere. Fibronectin binding protein, haemolysin, and type VII secretion system (T7SS) were found to be related to skin infections. Alpha-haemolysin is a heptameric pore-forming β-barrel toxin that is well known as the main virulence factor affecting haemolysis [46], both in humans and animals [47]. This haemolysin causes the main harmful effects by breaking down and damaging cells, especially in humans, including red blood cells, skin cells, and blood vessel cells [48]. Particularly, epithelial cells and keratinocytes were reported as alpha-haemolysin-susceptible cells [49,50], which might be damaged by this haemolysin type during skin infection caused by staphylococci. Moreover, this type of haemolysin also induced inflammation-related mediators [51], which help to aggravate the skin infections. Beta-haemolysin is mentioned as a Mg^2+^-dependent sphingomyelinase, which degrades sphingomyelin [52], that was found to be produced by both *S. aureus* [47] and *S. pseudintermedius* [53]. According to a previous report in mice, this haemolysin affected the epidermal growth factor, which in turn caused skin inflammation [54]. The main effect of beta-haemolysin is red blood cell haemolysis; however, this toxin has other pathogenicity, particularly inflammation-related mediators’ production stimulation [55]. Moreover, this haemolysin type is also indicated as a crucial factor in skin colonisation and induced skin cell damage [56]. Fibronectin binding protein, particularly type A, was recognised as an important factor in the initial adhesion of staphylococci, especially *S. aureus,* for the infection [57], and it might be considered a virulence factor inducing bacteria to attach to skin cells [58]. Lastly, the T7SS was also reported as a virulence factor associated with skin abscessation caused by staphylococci [59].

Commonly, MRSA harboured the *mecA* gene encoding PBP2a, leading to methicillin resistance in staphylococci [60]. Moreover, the *mecC* was reported as a mutated *mecA* gene that was linked to transmission from animals to humans [61]. In addition, the development of antibiotic resistance in MDR staphylococci was attributed to the activation of the efflux pump observed in MDR isolates [62]. For tetracycline resistance, *tet* genes, encoding membrane-associated efflux proteins and ribosome-protective proteins, were related to the resistance; *tetK* and *tetL* were mostly found in staphylococci [35]. Furthermore, *tetM* and *tetK* [35] were strongly found in staphylococci isolated from animals [36]. In *Staphylococcus* spp., approximately 25% of their genome was defined as MGEs [63]. Resistance genes of staphylococci, particularly *S. aureus*, were mentioned in the increased MGEs and horizontal gene transfer [63,64]. Several MGEs were found in *S. aureus*, including transposons, transposon-like elements, and insertion sequences [65]. Several macrolide resistance genes were identified in staphylococci isolated from many sources, and some of them were revealed through association with MGEs [66]. The *mefE* gene was previously found as one of the macrolide resistance genes that was found in the MGE event in staphylococci [66]. Remarkably, bacteriophages, plasmids, pathogenicity islands, transposons, and cassette chromosomes were referred to as the horizontal gene transfer events mostly found in staphylococci [67]. Interestingly, the application of antibiotics in animal farming was mentioned as the main source of antibiotic resistance gene spreading, which promotes a high chance of MGEs and horizontal gene exchange occurrence [68,69]. Nevertheless, the occurrence of MGEs and horizontal gene exchange in pet animals is less and should be further studied. For horizontal gene transfer, tetracycline resistance genes were found to have a high prevalence of antibiotic resistance genes in whole genomes. Some transposons, such as Tn6079, were found to have the ability to carry tetracycline resistance genes, especially *tetM* and *tetL*, in human infants’ gut bacteria [70]. Previous studies mentioned that some transposons, such as Tn5406 and Tn916, were associated with macrolide and tetracycline resistances in *S. aureus*, respectively [71,72]. For *S. pseudintermedius*, the transfer of transposons can be found in this species, the same as the other staphylococci [72]. The horizontal gene transfer occurrence in *S. pseudintermedius* was found to show lower transfer frequencies of plasmid than other species, particularly *S. aureus*; however, there was no difference between these species in transposon transfer frequencies [73]. A previous study mentioned that a phage belonging to the Triavirus contained the lysin gene, which could be applied for specific anti-MRSA bacteriophage- and phage lysin-based therapeutic approaches [74]. The application of phage treatment in multidrug-resistant staphylococci is particularly fascinating. Moreover, the application of phage mixtures has proven to be an alternative method in combating multidrug-resistant bacteria [75].

Whole genome sequences of all the MDR isolates should be determined as one of the study’s limitations. However, *S. aureus* WU1-1 and *S. pseudintermedius* WU48-1 were selected based on their multidrug-resistant profile. Further research should be conducted on the relationship between staphylococci isolated from animals and their owners. In addition, in vivo/ex vivo studies to provide insight into the pathogenesis of both *S. aureus* and *S. pseudintermedius* in animals should be investigated, aimed at designing new therapeutic strategies.

## 4. Materials and Methods

### 4.1. Ethical Approval

All procedures conducted on animals were approved by the Ethics Scientific Committee of Walailak University, Nakhon Si Thammarat, Thailand (Ref No. WU-ACUC-65081). The manipulation, restraint, and lesion swabbing of the studied animals were performed by licensed veterinarians. All the experiments were performed under the regulation of biosafety for scientific experiments (Ref. No. WU-IBC-66-019) of Walailak University, Nakhon Si Thammarat, Thailand.

### 4.2. Isolation and Identification of Bacteria

A total of 56 samples were collected from animals from several animal hospitals and shelters located in Nakhon Si Thammarat province, Southern Thailand, during August to December 2023. The swab samples were collected from superficial pyoderma in 42 dogs and 14 cats. The characteristics of sample collection sites are any skin lesions with pus that were present on the skin of dogs or cats. The area surrounding the pyoderma was cleaned with 75% medical alcohol and normal saline solution. A sterile cotton swab was swabbed on the pyoderma lesion. The swab samples were placed in a sterile glass tube containing sterile phosphate buffer saline solution (PBS) and immediately transported at room temperature to the laboratory. Then, 100 µL of the suspension was spread on mannitol salt agar (MSA) plates (HiMedia, India). The inoculated plates were incubated at 37 °C for 18–24 h, and staphylococcal-like colonies containing yellow and pink colonies were further grown on tryptic soy agar (TSA) (Difco, Claix, France). Then, the species of isolates were further identified by matrix-assisted laser desorption/ionisation time-of-flight mass spectrometry (MALDI-TOF MS) at the Office of Scientific Instrument and Testing, Prince of Songkla University. *Staphylococcus* confirmed isolates were kept at −80 °C in tryptic soy broth (TSB) (Difco, Claix, France) containing 25% glycerol until used. Moreover, *S. aureus* ATCC25923 and *S. aureus* DMST4775 were used as the reference strains.

### 4.3. Antibiotic Susceptibility

Antibiotic susceptibility of the staphylococcal isolates was determined by disc diffusion assay according to Clinical and Laboratory Standards Institute (CLSI) M100 30ed guidelines [76]. Briefly, 3–5 colonies of each staphylococcal isolate cultivated on TSA were adjusted in 0.85% normal saline solution (NSS) to obtain a 0.5 McFarland standard. Then, the suspension was spread on Mueller Hinton agar (MHA) (HiMedia Laboratories, Mumbai, India) using sterile cotton swabs. Furthermore, 7 antibiotic classes with 10 standard antibiotic discs (Oxoid, Hampshire, UK), including penicillin G (PEN), ampicillin (AMP), methicillin (MET), erythromycin (ERY), chloramphenicol (CHL), ciprofloxacin (CIP), kanamycin (KAN), gentamicin (GEN), doxycycline (DOX), and trimethoprim (TRI), were used, and then the plates were incubated at 37 °C for 16–18 h. The investigations were performed in triplicate for all tested isolates. The inhibition zone of each antibiotic was measured using a vernier calliper and compared to the standard diameters recommended in the CLSI M100 30ed guideline [76]. The inhibition zone was measured as the diameter length in millimetres at which the tested antibiotics inhibit the visible growth of bacteria. The results were interpreted as sensitive (S), intermediate (I), and resistant (R), and the isolate that presented resistance to at least three antimicrobial classes was defined as multidrug resistant (MDR).

### 4.4. Bacterial DNA Extraction

Genomic DNA of the antibiotic-resistant staphylococci, including *S. aureus*, *S. pseudintermedius*, *S. sciuri*, and *S. epidemidis*, was extracted using a commercial DNA extraction kit (ZymoBIOMICS DNA Miniprep kit, Zymo Research Corp., Irvine, CA, USA) according to the manufacturer’s protocol. Briefly, a loop full of fresh colonies of each bacterium was suspended in 750 µL of the lysis solution, aliquoted in ZR-BashingBead™ lysis tubes (Zymo Research Corp., Irvine, CA, USA). The samples were vortexed at high speed for 40 min and centrifuged at 100,000× *g* for 1 min. Cell-free supernatant was transferred to the Zymo-Spin™ III-F filter, centrifuged at 8000× *g* for 1 min. The filter membrane containing the spin column was placed in a new collection tube, and then DNA blind buffer was added to the spin column once again. DNase/RNase-free water was added to the centre of the membrane and then incubated at room temperature. The spin column was centrifuged at 10,000× *g* for 1 min for genomic DNA collection in a new 1.5-mL microcentrifuge tube. The sample was stored at −20 °C until used for further steps.

### 4.5. Detection of Antibiotic Resistance Genes

The representative isolates of antibiotic-resistant *S. aureus*, *S. pseudintermedius*, *S. sciuri*, and *S. epidermidis* were chosen to detect the antibiotic-resistant genes using specific primers (Table 7) by polymerase chain reaction (PCR). Briefly, the extracted DNA (5 µL) was added to a PCR tube containing 15 mL of Master-Mix (1 µL of 10 mM of each dNTP, 2.5 µL of 10X of *Taq* reaction buffer with MgSO_4_, 0.3 µL of 5 U/µL of *Taq* DNA polymerase, 0.2 µL of DMSO, and 1 µL of each forward and reverse primer) and 9 µL of DNase-RNase-proteinase-free water. The PCR conditions followed the previous studies [77,78,79,80,81,82,83]. Ten microlitres of the PCR product were mixed with 2 µL of DNA staining agent and run for 40 min at 135 V on a 1.5% agarose gel under a 0.5X TAE buffer. The positive suspected band was compared in size with the 100-bp DNA ladder marker. The agarose gel was observed under an ultraviolet illuminator.

### 4.6. Whole Genome Sequencing and Bioinformatics

Representative isolates, *S. aureus* WU1-1 and *S. pseudintermedius* WU48-1, were chosen to sequence their whole genome. The extracted DNA with the proper quality was used for the De Novo sequencing, and the obtained reads were further analysed. In brief, the short reads of each isolate were assembled using Velvet version 1.2.10, and the de Bruijn plot was performed to assess the k-mers overlapping relationship. Then, the contigs were aligned and assembled into scaffolds using GapFiller version 1.10 and SSPACE version 3.0, respectively. All assembled sequences were predicted gene coding regions using Prodigal version 2.6.3. The gene coding regions were further annotated for their function using BLAST (https://blast.ncbi.nlm.nih.gov/Blast.cgi, accessed on 1 February 2025), and the alignment result with an E-value < 0.00001 was the best-chosen result. The NR database was used to classify and confirm the species of the isolates. In addition, the comprehensive antibiotic resistance database (CARD) was used for determining the antibiotic resistance ontology and AMR gene detection. The virulence factor database (VFBD) was performed to curate information about virulence factors of bacterial pathogens. The whole genome sequence of *S. aureus* that matched several sources in the same MLST was additionally analysed for core genome single-nucleotide polymorphism (cgSNP) using tools in the Galaxy web server (https://usegalaxy.org.au, accessed on 15 February 2025). The Prokka tool was used to annotate the genome, and the Roary tool was used to generate the alignment of the core genes. Moreover, the Finds SNP sites tool was used to create the multi-FASTA alignment file. Then, the core gene sequences and SNP site were used to build a maximum-likelihood phylogenetic tree. The phylogenetic tree and SNP sequence were merged to display the final phylogenetic tree with SNP in core genomes using iTOL v.7 (https://itol.embl.de, accessed on 15 February 2025).

### 4.7. Statistical Analysis 

The data obtained were recorded in spreadsheets of Microsoft Excel 2021. All statistical analyses were performed on the R programming language version 4.3.1. In brief, descriptive statistics were applied to all the collected data. Moreover, staphylococci proportion between each animal species (dogs and cats) was further analysed using the chi-square test to determine the difference between these factors. All performed statistical tests were analysed under a 95% confidence interval, and a *p*-value < 0.05 was defined as a significant level.

## 5. Conclusions

This study revealed the prevalence of antibiotic-resistant staphylococci isolated from pyoderma in dogs and cats. Both *S. pseudintermedius* and *S. aureus*, the main causative agents of pyoderma, were detected. Staphylococcal isolates were greatly resistant to β-lactam and chloramphenicol, tetracycline, and aminoglycoside antibiotics, of which most isolates carried *blaZ*, *aph3*, and *tetM* genes. The MDR-*S. aureus* WU1-1 was matched to ST2990, while the MDR-*S. pseudintermedius* WU48-1 was a novel sequence type. Remarkably, SCCmec cassette genes were found in both isolates; however, the *mecA* gene was found only in MDR-*S. pseudintermedius* WU48-1. Genes in the antibiotic target alteration mechanism were frequently found in mobile gene transfer and horizontal gene transfer events. Both *S. aureus* WU1-1 and *S. pseudintermedius* WU48-1 genomes were inserted with Caudiviricetes bacteriophage DNA. The findings of these multidrug-resistant staphylococci, including antimicrobial resistance genes, mobile genetic elements, and horizontal gene transfer, might improve the understanding of gene transmission and serve as information for antibiotic resistance monitoring in veterinary medicine.

## Figures and Tables

**Table 1:** Prevalence of staphylococci isolated from canine and feline superficial pyoderma.

Animals	No. of Isolates	No. of Staphylococci	*p*-Value
Canine (*n* = 42)	75 (76.53%)	46 (46.94%)	0.93 ^ns^
Feline (*n* = 14)	23 (23.47%)	15 (15.31%)	
Total	98 (100%)	61 (62.24%)	

ns = no statistical significance (*p* ≥ 0.05).

**Table 2:** Species identification of staphylococci and non-staphylococci microorganisms isolated from the skin infection of dogs and cats.

Groups of Microorganisms	No. of Isolates	Percentage (%)
1. Coagulase positive staphylococci		
*Staphylococcus aureus*	5	5.10%
*Staphylococcus delphini*	1	1.02%
*Staphylococcus intermedius*	13	13.26%
*Staphylococcus pseudintermedius*	3	3.06%
2. Coagulase negative staphylococci		
*Staphylococcus arlettae*	1	1.02%
*Staphylococcus cohnii*	8	8.16%
*Staphylococcus epidermidis*	2	2.04%
*Staphylococcus felis*	2	2.04%
*Staphylococcus pasteuri*	3	3.06%
*Staphylococcus saprophyticus*	13	13.26%
*Staphylococcus sciuri*	8	8.16%
*Staphylococcus simulans*	2	2.04%
3. Non-staphylococci		
*Brevibacterium casei*	7	7.14%
*Micrococcus luteus*	11	11.22%
*Enterococcus faecalis*	1	1.02%
*Enterococcus faecium*	2	2.04%
*Enterococcus hirae*	2	2.04%
*Rothia nasimurium*	2	2.04%
4. Yeast		
*Candida tropicalis*	8	8.16%
5. No organism identification	4	4.08%
**Total**	**98**	**100%**

**Table 3:** Antibiotic susceptibility profiles of staphylococci isolated from dogs and cats (*n* = 61).

Antibiotics		Susceptibility (*n* = 61)			Reference Strains (*S. aureus*)	
Class	Name	S	I	R	ATCC25923	DMST4775
Aminopenicillin	Penicillin G	39	4	18	S	S
	Ampicillin	47	0	14	S	S
	Methicillin	59	1	1	S	S
Macrolide	Erythromycin	36	12	13	S	S
Phenicol	Chloramphenicol	53	1	7	S	S
Fluoroquinolone	Ciprofloxacin	59	0	2	S	S
Aminoglycosides	Kanamycin	52	1	8	S	S
	Gentamicin	60	1	0	S	S
Tetracycline	Doxycycline	49	0	12	S	S
Antifolate	Trimethoprim	41	16	4	S	S

S: sensitive, I: intermediate, R: resistant.

**Table 4:** Antibiotic resistance patterns of MDR strains of isolated staphylococci in dogs and cats (*n* = 11).

MDR Pattern	No. of Drug Class	No. of Isolates
PEN-ERY-DOX	3	1 (9.09%)
PEN-AMP-KAN-DOX	3	6 (54.55%)
PEN-AMP-DOX-TRI	3	2 (18.18%)
PEN-AMP-CIP-KAN-TRI	4	1 (9.09%)
PEN-AMP-MET-ERY-CHL-CIP-KAN-DOX-TRI	7	1 (9.09%)
**Total**	**-**	**11 (100%)**

PEN = Penicillin; AMP = Ampicillin; MET = Methicillin; ERY = Erythromycin; CHL = Chloramphenicol; CIP = Ciprofloxacin; KAN = Kanamycin; DOX = Doxycycline; TRI = Trimethoprim.

**Table 5:** Detection of antibiotic-resistant genes in the clinical isolates of *S. aureus*, *S. pseudintermedius*, and *S. sciuri* from dogs and cats (*n* = 16).

Isolates	Antibiotic-Resistant Genes								
	Beta-Lactam			Trimethoprim	Aminoglycoside		Chloramphenicol	Tetracycline	
	*blaZ*	*mecA*	*aac*	*dfrK*	*aph2*	*aph3*	*cat1*	*tetK*	*tetM*
*S. aureus*									
WU1-1	+	-	-	-	-	+	-	-	-
WU7-2	+	-	-	-	-	+	-	-	-
WU13-1	+	-	-	-	-	+	-	-	-
WU20-1	+	-	-	-	-	-	-	-	+
WU52-2	+	-	-	-	-	+	-	-	-
*S. pseudintermedius*									
WU47-1	+	-	-	-	-	-	-	-	+
WU48-1	+	+	+	+	-	+	-	-	+
WU55-1	-	-	-	-	-	-	-	-	+
*S. sciuri*									
WU10-2	-	-	-	-	-	+	-	-	-
WU29-1	+	-	-	-	-	-	-	-	-
WU30-2	-	-	-	-	-	-	-	-	-
WU34-1	-	-	-	-	-	-	-	-	-
WU35-1	-	-	-	-	-	-	-	-	-
WU35-2	+	-	-	-	-	-	-	-	-
WU37-2	-	-	-	-	-	-	-	-	-
WU53-1	+	-	-	-	-	+	-	-	-
*S. epidermidis*									
WU24-1	+	-	-	-	-	-	-	-	-
WU15-1	+	+	-	-	-	-	-	+	-
No. of isolates	12	2	1	1	0	7	0	1	4
Percentage (%)	67	11	6	6	0	39	0	6	22

**Table 6:** Information on the whole genome characteristics of MDR-*S. aureus* WU1-1 and MDR-*S. pseudintermedius* WU48-1.

Feature	*S. aureus* WU1-1	*S. pseudintermedius* WU48-1
1. Information on NCBI database		
Assembly accession	GCA_037083895	GCA_037132735
2. Genome characteristics		
Genome size	2.8 Mbp	2.7 Mbp
G+C content of genome	32.5%	37.5%
3. Genes		
Total gene	2814	2625
Protein coding	2668	2475
Non-coding	1	1
rRNA	13	15
tRNA	59	58
Pseudogene	70	73
Others	3	3
4. Virulent factor annotation		
Capsule	16	1
Staphyloferrin A and B	19	1
Fibronectin binding protein	11	nf
Exotoxin	9	nf
Hemolysin (α, β, γ, and δ)	7	nf
Type VII secretion system	6	nf
Coagulation factors	4	nf
Leukocidin	3	2
Leukotoxin	2	nf
Other binding proteins	2	nf
Aureolysin	1	nf
Autolysin	1	nf
Exfoliative toxin	1	nf
Others	46	1
5. Antimicrobial resistance gene annotation		
Antibiotic efflux	88	75
Antibiotic target alteration	67	64
Antibiotic target protection	7	9
Antibiotic inactivation	6	16
Antibiotic target replacement	5	6
6. Resistance of common antibiotic group		
Macrolide group	27	25
Tetracycline group	21	25
Fluoroquinolone group	18	14
Beta-lactam group	14	19
Aminoglycoside group	7	13
Sulphonamide group	1	1
7. Genes related to methicillin resistance		
*mecA*	nf	Found in genome
*mecC*	Found in genome	nf
*mecR1*	Found in genome	Found in genome
*mecI*	Found in genome	nf

nf: not found in the genome.

**Table 7:** Details of primers used for antibiotic resistance in isolated staphylococci.

Resistant Genes	Target Gene	Primer Sequence	Size (bp)	Ref.
Beta-lactam	*blaZ*	F: 5′-TGACCACTTTTATCAGCAACC-3′	240	[77]
		R: 5′-GCCATTTCAACACCTTCTTTC-3′		
	*mecA*	F: 5′-AAAATCGATGGTAAAGGTTGGC-3′	533	[78]
		R: 5′-AGTTCTGCAGTACCGGATTTGC-3′		
Trimethoprim	*dfrK*	F: 5′-GCTGCGATGGATAAGAACAG-3′	214	[79]
		R: 5′-GGACGATTTCACAACCATTAAAGC-3′		
Aminoglycoside	*aph2*	F: 5′-GAAGTACGCAGAAGAGA-3′	491	[80]
		R: 5′-ACATGGCAAGCTCTAGGA-3′		
	*aph3*	F: 5′-AAATACCGCTGCGTA-3′	242	[80]
		R: 5′-CATACTCTTCCGAGCAA-3′		
Chloramphenicol	*cat1*	F: 5′-GCGAACGAAAAACAATTGCA-3′	748	[81]
		R: 5′-TGAAGCTGTAAGGCAACTGG-3′		
Tetracycline	*tetK*	F: 5′-GTAGCGACAATAGGTAATAGT-3′	360	[82]
		R: 5′-GTAGTGACAATAAACCTCCTA-3′		
	*tetM*	F: 5′-AGTGGAGCGATTACAGAA-3′	158	[83]
		R: 5′-CATATGTCCTGGCGTGTCTA-3′		

## Data Availability

The original contributions presented in this study are included in the article. Further inquiries can be directed to the corresponding author. The article contains DNA sequence information. The nucleotide sequence data were submitted to GenBank (NCBI). The draft whole genomes of MDR-*S. aureus* WU1-1 and MDR-*S. pseudintermedius* were assigned as accession numbers: GCA037083895 and GCA037132735, respectively.
